# Allostatic load and physiological responses to work stress: an integrative review

**DOI:** 10.47626/1679-4435-2023-945

**Published:** 2024-02-16

**Authors:** John Camillo Garcia, Anibal Arteaga

**Affiliations:** 1 Corporación Universitaria Remington, Grupo de Investigación en Salud Familiar y Comunitaria, Facultad de Ciencias de la Salud, Medellín, Antioquia, Colombia

**Keywords:** occupational stress, allostasis, professional burnout, estrés laboral, alostasis, agotamiento profesional

## Abstract

Assessing the allostatic load of workers in the context of COVID -19 is of vital importance to elucidate the physiological responses to social and work stress. This is an integrative review of the literature including seven established steps: 1) identification of the topic and the guiding question; 2) definition of MeSH terms and search equations; 3) search in databases following defined criteria; 4) data collection according to inclusion criteria; 5) evaluation of the studies included in the integrative review; 6) discussion of results; and 7) presentation of the review/synthesis of knowledge. Seventeen studies were included, of which 15 were cross-sectional observational studies and two were longitudinal studies. Heterogeneity in the measurement of allostatic load was the common denominator of the studies. Allostatic load is mentioned in all of them as a parameter of measurement, but they measured it diferently; therefore, the relationship between burnout, work environment, and allostatic load, although positive in most studies, was highly variable. In conclusion, it is necessary to conduct studies that combine both biological markers and clinimetric tests, trying to standardize the batery of tests of allostatic load, so that the correlation with work stress is significant and reliable. Similarly, allostatic load requires a systemic and interdisciplinary approach, since this condition puts chronic stress on all organs and physiological compensation mechanisms. Therefore, the allostatic load invites to a comprehensive care of people, considering the work, social, psychological, and biological domains.

## Abstract (es)

Valorar la carga alostática de los trabajadores en el contexto de la Covid-19 es de vital importancia para dilucidar las respuestas fisiológicas al estrés social y laboral. Esta es una revisión integrativa de la literatura, de siete pasos establecidos: 1) identificación del tema y de la pregunta orientadora; 2) definición de términos MeSH y ecuaciones de búsqueda; 3) búsqueda en bases de datos siguiendo criterios definidos; 4) recopilación de datos acorde a criterios de inclusión; 5) evaluación de los estudios incluidos en la revisión integradora; 6) discusión de resultados; 7) presentación de la revisión/síntesis de conocimientos. Se incluyeron 17 estudios, de los cuales 15 fueron estudios observacionales transversales y dos estudios longitudinales. La heterogeneidad en la medición de la carga alostática fue el común denominador de los estudios. En todos se menciona la carga alostática como parámetro de medición. Sin embargo, todos la midieron de formas diferentes, por lo que la relación entre desgaste ocupacional y carga alostática, aunque positiva en la mayoría de los estudios, fue muy variable. Se concluye que es necesario realizar estudios que combinen tanto los marcadores biológicos como las pruebas clinimétricas, tratando de estandarizar la batería de exámenes de la carga alostática, para que la correlación con estrés laboral sea significativa y confable. De igual forma, la carga alostática requiere un abordaje sistémico e interdisciplinario, dado que se ponen en tensión crónica todos los órganos y mecanismos de compensación fisiológicos. Por lo tanto, la carga alostática hace una invitación a un cuidado integral de las personas, desde los ámbitos laboral, social, psicológico y biológico.

## INTRODUCTION

The SARS-CoV-2 pandemic (coronavirus 2019 or COVID-19) has posed unprecedented challenges to health systems worldwide, revealing major deficiencies in countries’ preparedness to similar health phenomena.^1^ To contain the virus, people were required to drastically change their lifestyle, in an effort to flaten the epidemic curve,^2^ so as to allow for collapsed health systems to gain time to respond to an emerging and highly contagious virus. These dramatic lifestyle changes, the presence of an invisible microorganism, uncertainty, fake news,^3^ worsening of socioeconomic problems,^4^ precariousness of some health systems. and surrounding fear represent stress factors for the world population.

COVID -19 seems to be the answer that justifies all problems; however, although it clearly has exacerbated some of them and generated new ones, it is insufficient as a causal response. One of these complex problems exacerbated during the pandemic was work stress, in which burnout syndrome consists of the highest level of decompensation between work stressors and individual adaptive capacities.

Therefore, it can be stated that this is a longstanding problem that in ancient times was known as Elijah’s fatigue but has only gained atention only recently, being acknowledged as a disease in the current International Classification of Diseases (ICD-11),^5^ which defined burnout as “a syndrome conceptualized as a result of chronic stress in the workplace that has not been successfully managed.

It is characterized by three dimensions: 1) feelings of energy depletion or exhaustion; 2) increased mental distance from one’s job, or feelings of negativism or cynicism related to one’s job; and 3) sense of ineffectiveness and lack of accomplishment. Burnout refers specifically to phenomena in the occupational context and should not applied to describe experiences in other areas of life.”^5^ However, this strictly occupational perspective ignores that there are social, economic, familiar, and even geopolitical, conditions that may be closely related to the development of burnout, as observed by other authors.^6^ Therefore, a limited point of view determines limited management and prevention approaches.

Currently, one out of five workers in Colombia suffers from burnout syndrome, professional burnout, or work fatigue.^7^ Recent studies conducted during the pandemics show an incidence of nearly 60% in healthcare professionals.^8^ Some authors^9^ have studied work-related biomarkers of chronic stress, specifically cortisol and norepinephrine. However, these studies were not able to find a direct relationship between work stress, its severity, and the somatic or neuroendocrine expression of the disease. There seems to be a resilience to stress, or that cortisol and norepinephrine are insufficient to explain the phenomenon, possibly requiring more complex measures to establish a relationship of stress and occupational burnout with physiological changes.

From this perspective, the theory of allostasis gains value, since it consists of the adaptation process of living beings. The problem lies in the fact that, in chronic states of adaptation to stress, there is such a great activation that the system remains loaded, i.e., although stress stimuli were extinguished, the body continues atempting to adapt to stress, a phenomenon named allostatic load.^10^

In this same sense, according to Hintsa et al.,^11^ it is possible to study the stress generated by burnout thorough allostatic load, since there is a direct relationship between increased biochemical and anthropometric markers and presence and severity of burnout syndrome. However, some studies^12^ observed that some individuals do not show increased biochemical and physical markers but were positive for occupational burnout or stress. Some authors named it physiological resilience to stress; nonetheless, in some circumstances this resistance to stress may be lost, causing allostatic load.^13^ Therefore, it is necessary to explore the relationship between allostatic load, method for assessing allostatic load, and work stress or burnout syndrome.

## METHODS

This is an integrative literature review, a technique that, according to Torraco,^14^ consists of the process of searching, critiquing, and synthesizing literature on a topic or body of knowledge in an integrated way such that new frameworks and perspectives on the topic are generated, through a rigorous and systematic search. In this sense, the present review followed seven established steps: 1) identification of the topic and the guiding question; 2) definition of MeSH terms and search equations; 3) search in databases following defined criteria; 4) data collection according to inclusion criteria; 5) evaluation of the studies included in the integrative review; 6) discussion of results; and 7) Presentation of knowledge review/synthesis. The guiding question was: what is the relationship between allostatic load and burnout syndrome in adults?

**Figure 1:**
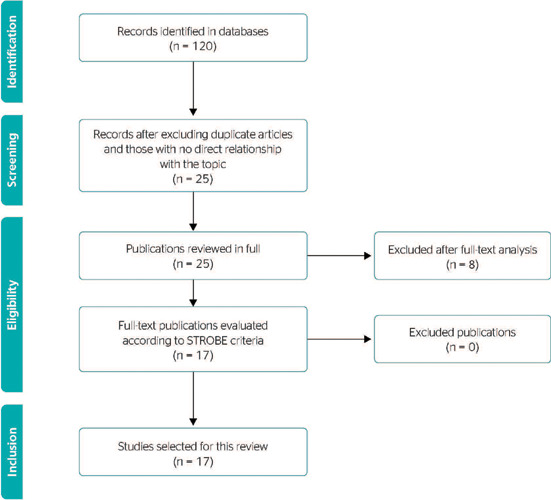
Document filtering chart.

Data search for complete scientific articles available was conducted in the following databases: PubMed, Scopus, EMBASE, SciELO, Biblioteca Virtual en Salud (BVS), Nature Journals, and Taylor and Francis, using the descriptors “alostasis”; “cortisol”; “eje hipotálamo hipófisis suprarrenal (HHS)”; “eje hipotalámico-pituitario-adrenal (HPA)”; “respuesta inmune”; “burnout”; “personal de salud”. Search equations were formulated with then operators “OR and AND,” allowing for multiple combinations, such as personal de salud AND burnout AND alostasis OR Cortisol OR Eje HPA OR Respuesta inmune. The filters applied were “full text available,” writen in English, Spanish, and Portuguese, date of publication from 2007 and 2021. Theses, dissertations, monographs, narrative reviews, leters to the editor, and editorials were excluded. Of the 120 results found, 17 articles were selected, which, afer reading of titles, abstracts, and full texts, met the inclusion criteria of this study. The process of article search and selection is described in Figure 1.

To assess the quality of the selected articles, two authors used the STROBE,^15^ which contains 22 items. Two authors evaluated each article and assigned 1 point for each present element, and 0 point for each absent one. When authors’ assessments did not coincide, all authors discussed and resolved the disagreement. All articles included in this review scored at least 20 points for the items evaluated.

## RESULTS

Of the 17 studies included, 15 were cross-sectional observational studies, and two were longitudinal studies. For the analysis of results, studies were classified according to measurement of allostatic load or biomarkers. Heterogeneity in the measurement of allostatic load was the common denominator of the studies. Allostatic load is mentioned in all of them as a parameter of measurement, although they measured it differently; similarly, the association of allostatic load biomarkers with occupational and psychosocial stress scales varied between the studies. Table 1 describes the biomarkers used in each study, the occupational or psychosocial tests or scales used, and the relationship between them.

**Table 1:** Studies analyzed, allostatic load parameters, and their relationship with occupational and psychosocial stress tests

Author	Year	Biomarkers evaluated	Stress tests	n
**Langelaan et al.** ^12^	**2007**	**SBP; DBP; BMI; CRP; HDL; HbA1C; glucose; cholesterol; WHR**	**MBI-GS**	**290**
Bellingrath et al.^16^	2009	C; E; NE; DHEA; WHR; HbA1c; HDL, cholesterol; SBP; DBP; TNF-a; CRP; fibrinogen; D-dimer; percent body fat, triglycerides; glucose	Effort-Reward Imbalance; Vital Exhaustion Questionnaire; MBI-EE	104
**Näswall et al.** ^17^	**2012**	**SBP; DBP; HR; WHR; lung function; HDL; LDL; HbA1C; salivary cortisol**	**Job Insecurity Score**	**1,359**
Juster et al.^18^	2011	C; DHEA-S; CRP; fibrinogen; insulin; Hb1AC; albumin; creatinine; amylase; HDL; total cholesterol; triglycerides; SBP; DBP; WHR	Trier Social Stress test; MBI-GS; The 22-item Beck Depression Inventory	30
Juster et al.^19^	2013	DBP, SBP; HR variability; insulin; HDL; LDL; triglycerides; CRP; TNF-a; IL-6; BMI; WHR; C	JCQ; Beck Anxiety Inventory; DCS model	199
**Ota et al.** ^20^	**2015**	**C; DHEA**	**Effort-Reward Imbalance;**	**115**
**Dich et al.** ^21^	**2015**	**SBP; DBP; BMI; insulin; glucose; HDL; LDL; triglycerides; CRP; IL-6.**	**Job Content Instrument**	**7,007**
Hintsa et al.^11^	2016	DBP, SBP; HDL; LDL; triglycerides; HB1AC; insulin; homocysteine; CRP; BMI; waist-height ratio; DHEA	MBI-GS; GHQ12; Beck Depression Inventory	8,028
Chandola & Zhang^22^	2018	Insulin-like growth factor; creatinine; DHEA-S; fibrinogen, CRP, HDL; cholesterol; triglycerides, HbA1c, HR, SBP; DBP; WHR	Job Quality Interview	244
Coronado et al.^23^	2018	Waist-height ratio; SBP; DBP; HbA1C; HDL; fibrinogen; triglycerides; # leukocytes; HR; C; insulin growth factor; PEF	Efort-Reward Imbalance	2,663
Rosemberg et al.^24^	2019	SBP; DBP; BMI; HR; WHR; HDL; HbA1C; CRP; C	JCQ; SF-12	49
D’Alonzo et al.^25^	2019	SBP; DBP; BMI; WHR; cholesterol; HDL; HbA1C; triglycerides; CRP	PSS; HWSSS	59
Esser et al.^26^	2019	TSH; CRP; leukocytes; triglycerides; LDL; HDL; homocysteine; creatinine; Hb1AC; DBP; DBP; HR; BMI	WAI	151
Peng et al.^27^	2021	Clinimetric criteria, without biomarkers	Kelner Symptom Questionnaire; SSRS	3,590
**Kerr et al.** ^28^	**2021**	**C; DHPA; testosterone; estradiol; progesterone; CRP; IL-6; fibrinogen; tumor necrosis factor-alpha; albumin; triglycerides; HDL; LDL; Hb1AC; insulin; creatinine; BMI; WHR**	**Bem Sex Role Inventory; 26 Item JCQ; The 17-item Effort-Reward Imbalance at Work Questionnaire; Beck Depression Inventory II; MBI-GS; Posttraumatic Stress Disorder Civilian Checklist**	**218**
López-Pumar et al.^29^	2021	SBP; DBP; BMI; WHR; leukocytes; glucose; creatinine; cholesterol; HDL; LDL	Stress Vulnerability Scale; Seppo Aro Stress Symptom Scale; Fuster-BEWAT score	142
Békési et al.^30^	2021	Fava’s clinimetric approach, without biomarkers.	Kelner Symptom Questionnaire; PHS-WB	228

The studies highlighted in bold describe no association between allostatic load and work stress or burnout (high allostatic load, with hypocortisolemia). BMI = body mass index; C = cortisol; CRP = C-reactive protein; DBP = diastolic blood pressure; DCS = Demand-Control-Support; DHEA = dehydroepiandrosterone; DHEA-S = dehydroepiandrosterone-sulphate; E = epinephrin; GHQ12 = 12-item General Health Questionnaire; HbA1C = glycosylated hemoglobin; HDL = high-density lipoprotein; HR = heart rate; HWSSS = Hispanic Women’s Social Stressor Scale; IL-6 = interleukin 6; JCQ = Job Content Questionnaire; LDL = low-density lipoprotein; MBI-EE = Maslach Burnout Inventory Emotional Scale; MBI-GS = Maslach Burnout Inventory General Survey; NE = norepinephrine; PEF = peak expiratory flow; PHSWB = Public Health Surveillance Well-being Scale; PSS = Perceived Stress Scale; SBP = systolic blood pressure; SF-12 = 12-item Short Form Health Survey; SSRS = Social Support Rating Scale; TNF-a = tumor necrosis factor-alpha; WAI = Work Ability Index; WHR = waist hip ratio.

All studies that mention measurement of allostatic load as an index ranging from 0 to 10 followed the procedure proposed by Seeman et al.,^31^ in which calculation was based on risk quartiles and 10 biomarkers, with allostatic load representing the sum of risk quartiles for each biomarker in a given person.

Of the overall included studies, 70% reported an association between allostatic load and work stress, the remaining 30% found no relationship between allostatic load index and the psychometric tests and the working, social, and individual conditions evaluated. The studies that reported an association between allostatic load and work stress had in common the fact that all of them evaluated blood pressure and high-density lipoprotein (HDL) cholesterol, secondary markers of anthropometric measures, and the other biomarkers varied among the studies.

### PRIMARY BIOMARKERS OF ALLOSTATIC LOAD INDEX

Two studies^27,30^ did not use biomarkers to calculate the allostatic load index, but rather the Fava et al. index,^32^ adapted from the psychosocial index. The measurement of at least one primary biomarker of allostatic load (dehydroepiandrosterone [DHEA], cortisol, norepinephrine, and epinephrine) was present in 60% of investigations, excluding purely clinimetric studies. The main primary biomarker was cortisol, measured in 53% of the studies, but the measurement technique was diverse, both regarding the type of sampling (urine, hair, saliva, and blood) and period of the day. The clinical interpretation of results for cortisol was also multiple, whereas high levels are associated with stress, low cortisol levels were described in two studies,^18,33^ showing an association between high allostatic load index and symptomatic burnout with great levels of depersonalization.

Other primary markers used were DHEA (33%), norepinephrine, and epinephrine (6.6%). The typical markers used, as described by Seeman, were modified in the study by Esser et al.,^26^ which used TSH hormones as primary markers of hypothalamic pituitary adrenal (HPA) axis activity in the absence of thyroid medication, with statistically significant results the regarding correlation with chronic stress, which shows once more the complexity and the systematicity of the effects of stress on the body.

### SECONDARY BIOMARKERS OF ALLOSTATIC LOAD INDEX

All studies included at least on secondary marker, and the classical secondary biomarkers (high-density lipoprotein, low-density lipoprotein, glycosylated hemoglobin, systolic blood pressure, diastolic blood pressure, waist-hip ratio) were the most commonly used. It was not possible to describe the statistical significance of each parameter and its sensitivity regarding the presence of allostatic load, since, when reporting allostatic load, it is described as the sum of the parameters that are within the high-risk quartile, and there is no detailed analysis of each biomarker.

As alternative secondary markers, it is worth noting the variability of tests that were used and adapted to the index, such as insulin-like growth factor, leukocytes, albumin, progesterone, interleukin 6, creatinine, fibrinogen, tumor necrosis factor-alfa, insulin, C-reactive protein, and heart rate variability. None of the studies used exactly the same combination of markers and secondary tests, which starts revealing the variability in the allostatic load index. However, there was a trend to include at least one primary biomarker combined with at least four secondary ones.

### STRESS TEST

The most used work stress and occupational burnout in the comparison with biomarkers were the MBI-GS = Maslach Burnout Inventory General Survey (MBI-GS),^11,12,18,28^ the Job Content Questionnaire,^21,22,24,28^ and the Effort Reward Imbalance (ERI),^16,20,23,24^ all of which were applied in four studies, with a greater percentage of correlation between MBI-GS and allostatic load, since this survey was used in studies with a larger number of participants with a positive association with the allostatic load index. The ERI test had the second highest association with the allostatic load index; the other tests and their results were highly variable.

The Psychosocial Index (PSI)^34^ was used in two studies^27,30^ to replace clinical biomarkers, considering the following steps: criterion A consists of the identification of the stressful factor or contextual threat that triggered stress, i.e., uncontrolled, unpredictable, and long-lasting factors, e.g., loss of an important person, health loss, work and family conflicts, among others, keeping in mind that some everyday situations may exceed the coping skills of an individual and become stressful factors, the identification of the source of stress represents the first step. The second point in the criteria, or criterion B, is concerned with clinical manifestations, which may include psychological symptoms and impairment in social and occupational functioning. A semi-structured interview that describes the assessment of these two steps was developed and validated in English by Fava et al.,^35^ and applied with some modifications in the aforementioned studies.

## DISCUSSION

Characterization of allostatic load index has been carried out by two distinct approaches. One is concerned with the use of biomarkers that reflect physiological derangements; the other is a clinical approach targeted to the more severe end of the associated symptomatology, subsumed under the rubric of allostatic overload. Most studies included in this review is centered on the identification of biological markers, taking the works by Seeman et al.^31^ as a reference with regard to primary and secondary markers. Furthermore, some authors^36^ determined some biological markers of diseases resulting from tertiary mediators of an allostatic load condition. However, the biological perspective does not per se allow for a comprehensive understanding on allostatic load and overload, and related clinical phenomena. In this sense, a substantial contribution seems to have come from clinimetrics, i.e., the science of clinical measurements, to complement a comprehensive view of patients with adaptative responses to stress.

Other variables that were analyzed and found to be related to high allostatic load are age-associated frailty, negative experiences in childhood and adolescence,^37^ being a caregiver, comorbidities, substance abuse, and obviously a stressful work environment.^38^ In this sense, there is still a scarcity of studies comparing working conditions, work stress, social and individual stress, and their relationship with allostatic load.

This review made it possible to detail the great heterogeneity among the studies with regard to the type and number of parameters to considerer and to the methodologies used to measure them, both in terms of the type of biological sample and for the laboratory test used. Similarly, work stress was measured by different ways and with distinct instruments, which makes it even more difficult to establish a clear correlation between the methods. It bears emphasizing that the use of clinimetric tools may increase the number of people who can be subjected to the tests, through initial clinimetric screening and subsequent laboratory tests, especially in low-resource settings. A mixed approach of these methods performed concurrently, although more costly, has shown better results.^38^

As for working conditions, it is necessary to highlight that these are modifiable factors associated with work, social and gender inequalities,^39^ work under pressure,^40,41^ low professional and moral recognition, among others. If these conditions are triggering stressors for allostatic load, they should be object of specific interventions, such as work reorganization and management of employees’ stress, in order to prevent morbid consequences for the individual and close people.^42^ With regard to burnout syndrome and its relationship with allostatic load, results are still inconclusive, especially due to the already described heterogeneity: of the five studies that measured burnout syndrome specifically through the MBI-SS, three found a relationship between allostatic load index^11,16,18^ and burnout, whereas the other two did not.^12,28^

Nonetheless, other tests, such as the ERI, showed a direct correlation with burnout syndrome, emotional exhaustion, and depersonalization; therefore, its directly proportional relationship with allostatic load seems to be associated with work stressors that trigger physiological compensation to stress.^29^

## FINAL CONSIDERATIONS

It is necessary to conduct additional studies that analyze the impact of work stress, burnout syndrome, and allostatic load specifically on healthcare workers, who have experienced significantly increased levels of occupational burnout. The heterogeneity of the batery of tests to measure the allostatic load index should be a factor to consider when designing the studies, since the excessive variability of the tests performed may condition the variability of results and their reliability. Similarly, it is necessary to conduct studies that combine biological markers and clinimetric tests, so as to demonstrate the correlation between clinical and biological parameters.

It also bears highlighting that allostatic load requires an interdisciplinary approach, both of stressful daily situations and of sudden and unplanned ones, without forgeting that the consequences of allostatic load are systemic, puting stress on all organs and physiological compensation mechanisms. Therefore, allostatic load invites to a comprehensive care of people, considering the work, social, psychological, and biological domains.
